# Operational lifetimes of organic light-emitting diodes dominated by Förster resonance energy transfer

**DOI:** 10.1038/s41598-017-02033-3

**Published:** 2017-05-11

**Authors:** Hirohiko Fukagawa, Takahisa Shimizu, Yukiko Iwasaki, Toshihiro Yamamoto

**Affiliations:** Japan Broadcasting Corporation (NHK), Science & Technology Research Laboratories, 1-10-11 Kinuta, Setagaya-ku, Tokyo 157-8510 Japan

## Abstract

Organic light-emitting diodes are a key technology for next-generation information displays because of their low power consumption and potentially long operational lifetimes. Although devices with internal quantum efficiencies of approximately 100% have been achieved using phosphorescent or thermally activated delayed fluorescent emitters, a systematic understanding of materials suitable for operationally stable devices is lacking. Here we demonstrate that the operational stability of phosphorescent devices is nearly proportional to the Förster resonance energy transfer rate from the host to the emitter when thermally activated delayed fluorescence molecules are used as the hosts. We find that a small molecular size is a requirement for thermally activated delayed fluorescence molecules employed as phosphorescent hosts; in contrast, an extremely small energy gap between the singlet and triplet excited states, which is essential for an efficient thermally activated delayed fluorescent emitter, is unnecessary in the phosphorescent host.

## Introduction

Organic light-emitting diodes (OLEDs) have been intensively studied as a promising technology for mobile displays, televisions and solid-state lighting^1–3^. Following electron-hole recombination, two types of molecular excited states (the singlet and triplet excited states) are formed in a 1:3 ratio determined by quantum spin statics^4^. The emission mechanism along with new luminescence materials have been intensively studied with the goal of harvesting all excitons as emission^5, 6^. The internal quantum efficiency (IQE), defined as the number of photons generated per injected carrier, is limited to 25% in first-generation fluorescent OLEDs. Although the efficiency of fluorescent OLEDs can be enhanced by utilising triplet-triplet annihilation, the upper limit of their IQEs is thought to be 62.5%^7^. Second-generation phosphorescent OLEDs (PHOLEDs) containing iridium complexes were first proposed in 1999, and IQEs of nearly 100% were achieved in the following year^8, 9^. In the 2010s, IQEs of third-generation OLEDs employing thermally activated delayed fluorescence (TADF) materials reached nearly 100% without the use of heavy metals such as iridium^10^. Currently, it is not uncommon to see it is not uncommon to see IQEs of approximately 100% in OLEDs employing phosphorescent or TADF emitters^11–14^.

In addition to efficiency, long operational lifetime is also of great significance for practical applications. In recent years, many research groups around the world have worked to improve the operational lifetimes of OLEDs^15, 16^. The suppression of exciton-polaron annihilation has been identified as an effective way to improve the operational lifetimes of OLEDs. For example, the operational lifetime of an OLED employing a TADF emitter was significantly improved by introducing interlayers of an electron-injection material^15^, and a ten-fold increase in the lifetime of a blue PHOLED was achieved by employing a graded dopant concentration profile in a broadened emitting layer^16^. Although device architectures have been proposed for demonstrating operationally stable OLEDs, materials suitable for operationally stable OLEDs are not yet understood systematically. This gap in knowledge has become a bottleneck in the development of operationally stable blue PHOLEDs and operationally stable OLEDs using TADF emitters.

Configurations of emitting layers (EMLs) that enable operationally stable PHOLEDs have been proposed over the last few years^17–21^. Highly efficient and stable PHOLEDs have been demonstrated using two suitable hosts: an exciplex with a small energy gap between the singlet (S_1_) and triplet (T_1_) excited states (Δ*E*_ST_)^18, 21^; and a single TADF material consisting of donor and acceptor units^19, 20^. The PHOLED employing a single TADF material as the host is thought to be the ideal emitter configuration for low-cost, high-performance PHOLEDs because it significantly reduces the usage of costly phosphorescent dopants^19, 20^. An operationally stable PHOLED can be realised using a TADF material as the host because the electrically excited triplet excitons of the host, which are generally unstable^22^, are transferred rapidly to the dopant following Förster resonance energy transfer (FRET) via reverse intersystem crossing (RISC) from the T_1_ state to the S_1_ state. However, little is known about the parameters and/or phenomena that dominate the operational stabilities of PHOLEDs; thus, the molecular design of the ideal host material is also unclear. Developing a strategy for the molecular design of the host material is important not only to obtain operationally stable blue PHOLEDs, but also to reduce the use of phosphorescent dopants in LEDs of all colours.

In this paper, we show a clear relationship between the operational lifetime of PHOLED and the FRET from the host to the dopant. By analysing the device characteristics of several PHOLEDs utilising similar TADF materials as hosts, we found the operational lifetime of PHOLED to be almost proportional to the Förster resonance energy transfer rate (*k*_FRET_). To our knowledge, this is the first report of a systematic relationship between operational lifetime and energy transfer. The relationship between operational lifetime and energy transfer was observed only after employing similar TADF materials as phosphorescent hosts since almost all the excitons generated in the host can be transferred by the Förster process. These findings suggest that TADF materials with small molecular sizes are suitable host materials for operationally stable PHOLEDs. On the other hand, an extremely small Δ*E*_ST_, which is essential for efficient TADF emitters, was found to be unnecessary.

## Energy transfer process

**Figure 1:**
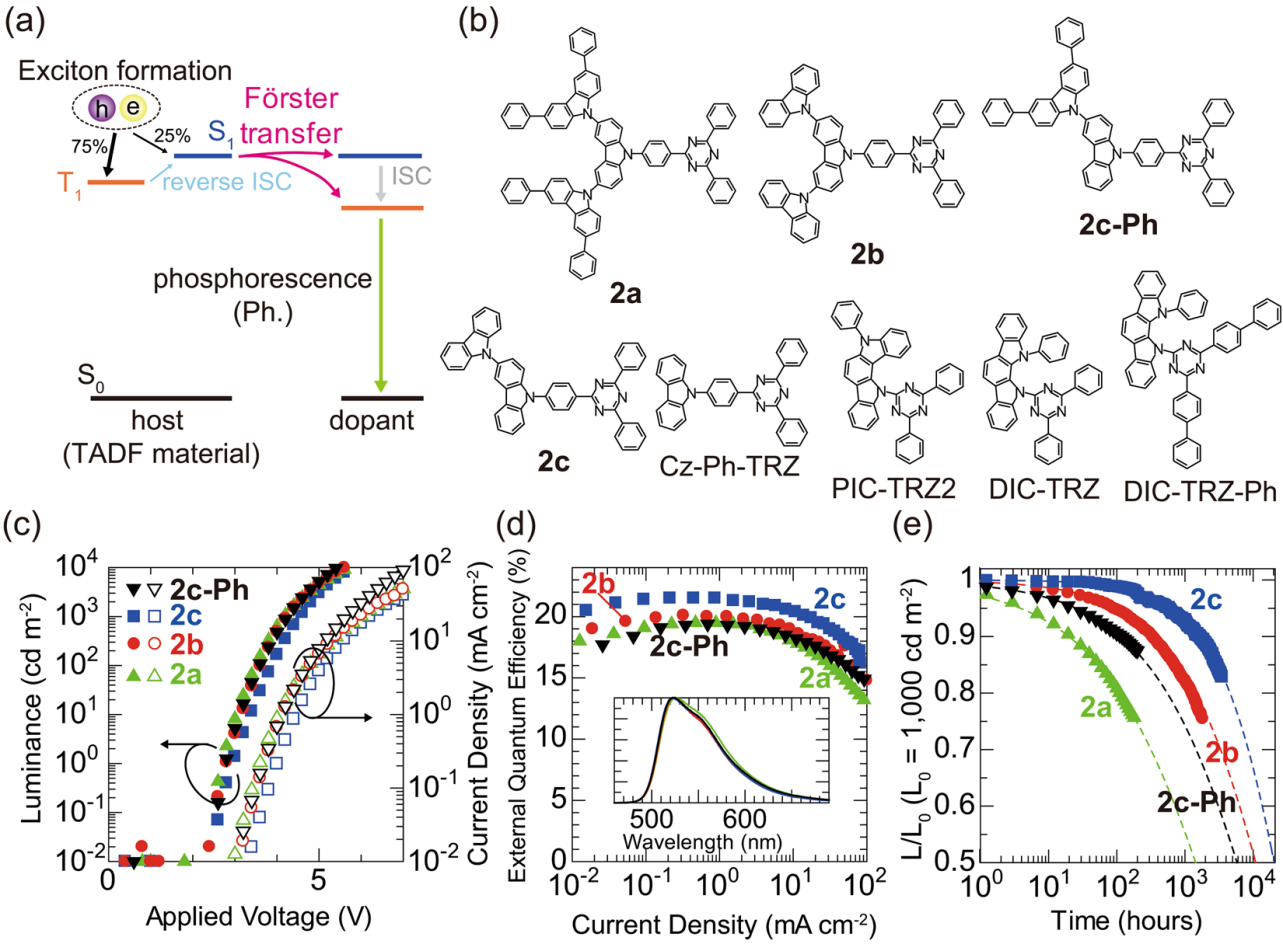
Schematic illustration of the energy transfer process and device performances of OLEDs. (**a**) Energy transfer process from the TADF host to phosphorescent emitter dopant. (**b**) Molecular structures of the host materials used in this study. (**c**) Luminance (left, filled symbols)- and current density (right, open symbols)-voltage characteristics of PHOLEDs. (**d**) EQE-current density curves of PHOLEDs. Inset: EL spectra of PHOLEDs. (**e**) Luminance-time characteristics for devices under a constant dc current with an initial luminance of 1,000 cd m^−2^.

Figure 1a shows the energy transfer diagram for the EML in a PHOLED employing a TADF material as host^20^. Singlet excitons formed by carrier recombination are simply transferred to the dopant following the FRET. Triplet excitons are also transferred via S_1_ states following the FRET; these host triplet excitons, which are generally unstable^22^, are transferred rapidly to the dopant following the FRET, resulting in the high operational stability of the PHOLED. The rapid energy transfer from host to dopant may also effectively suppress exciton-polaron annihilation^15, 16^. Thus, RISC in the TADF material and/or FRET from the host to the dopant are expected to be related to the extended operational lifetime. Although two known rate constants, the RISC rate constant (*k*_RISC_) and the *k*_FRET_, are related to energy transfer, the parameters that determine the operational lifetime remain unclear.

## PHOLED device structure and characteristics

In this study, we evaluated the characteristics of PHOLEDs utilising similar TADF materials as hosts to clarify the parameters that affect operational lifetime. Figure 1b shows the TADF materials used as hosts, which are categorised into two families. The first family contains **2a**, **2b** and **2c**, which consist of several carbazoles (s-Czs) and triazine; **2a** is 9′-[4-(4,6-diphenyl-1,3,5-triazin-2-yl)phenyl]-3,3″,6,6″-tetraphenyl-9,3′:6′,9″-ter-9H-carbazole, **2b** is 9′-[4-(4,6-diphenyl-1,3,5-triazin-2-yl) phenyl]-9,3′:6′,9″-ter-9H-carbazole and **2c** is 9-[4-(4,6-diphenyl-1,3,5-triazin-2-yl)phenyl]-3,9′-bi-9H-carbazole^14^. In addition to these TADF materials, we used 9-(4-(4,6-Diphenyl-1,3,5-triazin-2-yl)phenyl)-3′,6′-diphenyl-9H-3,9′-bicarbazole (**2c**-**Ph**) and 9-(4-(4,6-Diphenyl-1,3,5-triazin-2-yl)phenyl)-9H-carbazole (Cz-Ph-TRZ). **2c**-**Ph** shows TADF as above three materials, on the contrary, Cz-Ph-TRZ does not show TADF (see Supplementary Section 1). The reason why we used Cz-Ph-TRZ is that the effect of RISC on the operational lifetime can be observed since the molecular structures of Cz-Ph-TRZ are almost the same as those of **2c**. A clear difference in thermal behaviour was not observed between **2a**, **2c** and Cz-Ph-TRZ (see Supplementary Section 2). The second family contains PIC-TRZ2, DIC-TRZ and DIC-TRZ-Ph, which consist of indolocarbazole (ICz) and triazine; PIC-TRZ2 is 5,12-dihydro-12-(4,6-diphenyl-1,3,5-triazin-2-yl)-5-phenylindolo[3,2-a]carbazole^23^, DIC-TRZ is 2,4-diphenyl-6-bis(12-phenylindolo)[2,3-a] carbazol-11-yl)-1,3,5-triazine^24^ and DIC-TRZ-Ph is 2,4-dibiphenyl-6-bis(12-phenylindolo)[2,3-a]carbazol-11-yl)-1,3,5-triazine. The device configuration of the fabricated PHOLEDs was ITO/Clevios HIL 1.5/α-NPD/4DBTP3Q/host:Ir(mppy)_3_/TPBi/LiF/Al, where ITO is indium tin oxide, Clevios HIL 1.5 (supplied by Heraeus Holding GmbH) is the hole-injection layer, α-NPD is 4,4′-bis[N-(1-naphthyl)-N-phenyl-amino]biphenyl, 4DBTP3Q is N3,N3″′-bis(dibenzo[b,d]thiophen-4-yl)-N3,N3″′-diphenyl-[1,1′:2′,1″:2″,1″′-quaterphenyl]-3,3″′-diamine^25^, Ir(mppy)_3_ is *fac*-tris(3-methyl-2-phenylpyridinato-N,C2′-)iridium(III) and TPBi is 1,3,5-tris(N-phenylbenzimidazol-2-yl)benzene (see Supplementary Section 2).

Figure 1c shows the current density (*J*)–voltage (*V*)–luminance (*L*) characteristics of the PHOLEDs in which **2a**, **2b**, **2c** and **2c**-**Ph** were used as hosts. In these three PHOLEDs, the concentration of Ir(mppy)_3_ was 3 wt%, which is lower than the concentration used in conventional PHOLEDs. The *J*–*V* characteristics are almost independent of the host material. Thus, these hosts are ideal for evaluating the host-dependent device characteristics since the carrier balance and carrier recombination zone, which may affect the efficiency and operational lifetime, are similar in all PHOLEDs. The external quantum efficiency (EQE) versus *J* characteristics of all the tested PHOLEDs are shown in Fig. 1d. The PHOLED’s normalised luminance as a function of operating time is shown in Fig. 1e for an initial luminance of 1,000 cd m^−2^. Unlike the *J*–*V* characteristics, the EQE and operational stability of the PHOLEDs depended on the host. The EQE of the PHOLED using **2c** was the highest, although it was not greatly different from those of other PHOLEDs. Although the molecular structures of the hosts were similar, the PHOLEDs exhibited clear differences in operational lifetime. In contrast, the stabilities of the TADF materials alone are similar; the operational stabilities of the host-only OLEDs using **2a** and **2c**, in which the EMLs consist only of the TADF materials, were nearly the same (see Supplementary Section 3). The much shorter lifetimes of the host-only OLEDs compared to the PHOLEDs suggest that the energy transfer from the TADF host to Ir(mppy)_3_ effectively enhances the lifetime. The host-dependence of the PHOLED lifetime may result from differences in parameters related to energy transfer.

## Analysis of operational lifetime

We examined the factors affecting the operational lifetime of PHOLED. The device characteristics of all PHOLEDs are summarised in Table 1 along with some parameters related to *k*_RISC_ and *k*_FRET_. We used the device data for both host families (s-Czs and ICz families) for analysis because we needed as much data as possible to systematically understand the host-dependent PHOLED characteristics (see Supplementary Section 4). Here, we define the operational lifetime LT50 as the time for the luminance to decay to 50% of the initial luminance of 1,000 cd m^−2^ (see Supplementary Section 5)^26^. The fact that the lifetime of the PHOLED using **2c** was the greatest among the s-Czs host family suggests that the correlation between *k*_RISC_ and operational lifetime is poor since *k*_RISC_ should be proportional to exp(−Δ*E*_ST_/*k*_B_ *T*)^6, 27^. Although the EQE of the OLED using **2a** as an emitter is the highest among **2a**, **2b** and **2c** ^14^, the lifetime of PHOLED using **2a** as a host is the shortest. On the other hand, smaller molecular weight corresponded to longer operational lifetime (Table 1 and Fig. 1b). Since *k*_FRET_ is strongly correlated with the energy transfer distance, FRET, not the RISC process in the TADF host, is expected to dominate the PHOLED lifetime. Before the analysis of operational lifetime based on FRET, we must demonstrate that the effect of Dexter energy transfer on the operational lifetime is quite small. Evidence that the long LT50s, which were observed in several PHOLEDs in this study, are dominated by FRET can be seen in the short LT50 of PHOLED using Cz-Ph-TRZ (Table 1, see Supplementary Section 6). If the effect of Dexter energy transfer on LT50 is large, the LT50 of PHOLED using Cz-Ph-TRZ, the molecular size of which is smaller than that of **2c**, can be longer than that of the PHOLED using **2c** since Dexter energy transfer rate is also strongly correlated with the energy transfer distance^28^. Thus, it has been reconfirmed that the triplet up-conversion in TADF host is essential for long operational lifetime, and the LT50s in the PHOLED using TADF material as host is dominated by FRET^17, 19, 20^.

**Table 1:** Performances of PHOLEDs using several TADF hosts and parameters related to *k*_FRET_.

Host	PHOLED performances*		Δ*E*_ST_ (eV)	Parameters related to *k*_FRET_					*k*_FRET_ (10^8^ s^−1^)
	EQE (%)	LT50 (hours)		*Φ*_PL_ (%)	*τ*_PL_ (ns)	*R*_0_ (nm)	*R*_max_ (nm)	*R* (nm)	
**2a**	19.5	1,260	0.11^14^	67.5	6.5	2.72	1.51	2.28	4.42
**2b**	20.0	11,000^†^	0.19^14^	62.0	7.1	2.70	1.31	2.08	6.68
**2c**	21.5	20,000^†^	0.29^14^	65.3	6.5	2.72	1.22	1.99	10.0
**2c**-**Ph**	19.4	5,800^†^	0.25	61.6	6.7	2.69	1.42	2.19	5.10
Cz-Ph-TRZ	19.8	600^†^	0.36	—	—	—	—	—	—
PIC-TRZ2	22.0	10,500^†^	0.003^23^	50.6	87	2.24	1.02	1.79	0.43
DIC-TRZ	21.8	14,000^†^	0.14	32.7	52	2.06	0.93	1.70	0.60
DIC-TRZ-Ph	16.0	6,500^†^	0.13	26.2	24	1.88	1.29	2.06	0.24

*Measured at 1 mA cm^−2^ (corresponds to the luminance of approximately 1,000 cd m^−2^).

EQE: external quantum efficiency.

^†^Estimated lifetime by fitting curve^26^ (see Supplementary Section 5).

Δ*E*_ST_: Energy gap between singlet and triplet excited states (see Supplementary Sections 1 and 4).

*Φ*_PL_, *τ*_PL_: Photoluminescence quantum yield and radiation lifetime of host (see Supplementary Section 7).

*R*_0_: Estimated critical distance for the concentration quenching (Förster radius, see Supplementary Section 7).

*R*_max_: Maximum molecular radius of host calculated by using Gaussian 09.

*R*: Separation between the centres of the host and Ir(mppy)_3_.

*k*_FRET_: Estimated Förster resonance energy transfer rate from host to Ir(mppy)_3_.

**Figure 2:**
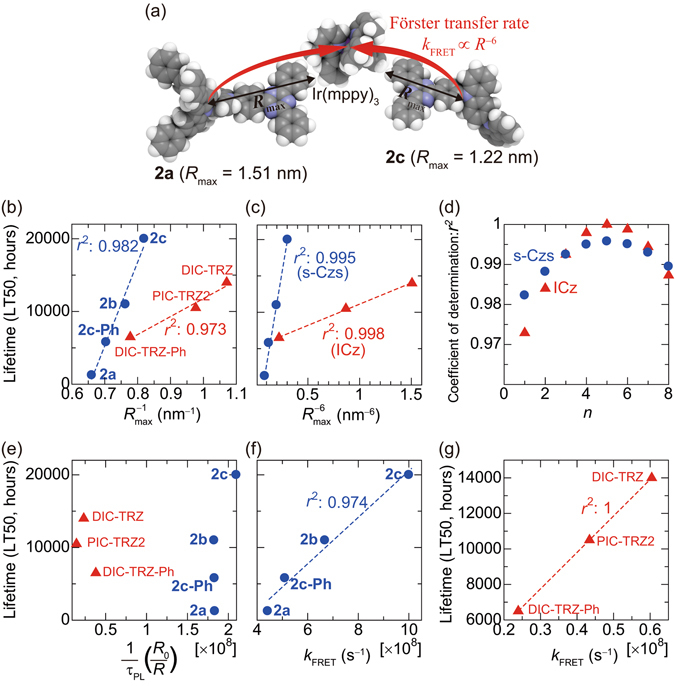
Schematic illustration of energy transfer distance and lifetime analysis. (**a**) Energy transfer distances from **2a** and **2c** to Ir(mppy)_3_. (**b**,**c**) Host-dependent PHOLED lifetime versus (**b**) inverse maximum molecular radius of host (${R}_{{\rm{\max }}}^{-1}$) and (**c**) ${R}_{{\rm{\max }}}^{-6}$; *r* ^2^ is the coefficient of determination for the linear approximation. (**d**) *r* ^2^ at each ${R}_{{\rm{\max }}}^{-n}$ as a function of a power index *n*. (**e**) PHOLED host-dependent lifetime versus 1/*τ*_PL_ × (*R*_0_/*R*). (**f**,**g**) PHOLED host-dependent lifetime versus *k*_FRET_ for the s-CZs host family (**f**) and the ICz host family (**g**).

We analysed the relationship between the operational lifetime of PHOLED and *k*_FRET_ from the donor (host) to the acceptor (dopant) Ir(mppy)_3_. Higher *k*_FRET_ is expected to correspond to longer operational lifetime because the excited states are generally unstable, and exciton-polaron annihilation can be suppressed by rapid energy transfer^15, 16^. *k*_FRET_ is represented by following the equation^29, 30^,

1 $${k}_{{FRET}}=\frac{1}{{\tau }_{PL}}{(\frac{{R}_{0}}{R})}^{6},\,{R}_{0}=\sqrt[6]{\frac{9000(\mathrm{ln}\,10){K}^{2}{{\rm{\Phi }}}_{PL}}{128{\pi }^{5}{N}_{A}{n}^{4}}\int {f}_{H}(\lambda ){\varepsilon }_{D}(\lambda ){\lambda }^{4}d\lambda },$$

where *τ*_PL_ is the intrinsic radiative decay of the host, *R*_0_ is the critical distance for the concentration quenching (Förster radius), *R* is the separation between the centres of the host and the dopant, *K* ^2^ is an orientation factor equal to 2/3 due to the random orientation of Ir(mppy)_3_^31^, *Φ*_PL_ is the quantum yield of the host, *N*_*A*_ is the Avogadro’s number, *n* is the refractive index of the medium (assumed to be 1.8), *λ* is the wavelength and $\int {f}_{H}(\lambda ){\varepsilon }_{D}(\lambda ){\lambda }^{4}d\lambda$ is the spectral overlap integral between the host’s PL *f*_H_(*λ*) and the dopant absorption *ε*_D_(*λ*). The calculated value of each parameter is summarised in Table 1 (see Supplementary Section 7). Although there are many parameters in Eq. 1, we first analysed LT50 with respect to *R*. The differences in LT50 among different PHOLEDs may be dominated by differences in *R*; *R*_0_ is almost independent of host as *Φ*_PL_ and *τ*_PL_ are similar among hosts, especially for the s-Czs host family (Table 1). A schematic of energy transfers from **2a** and **2c** to Ir(mppy)_3_ is shown in Fig. 2a. To simplify the analysis, we used a maximum molecular radius (*R*_max_) for the hosts instead of *R* for the following reasons: (1) the distance from the centre of Ir(mppy)_3_ to the host is host-independent due to the cubic shape of Ir(mppy)_3_; and (2) the principal difference in distance among hosts is the maximum molecular radius *R*_max_, which is estimated using Gaussian 09, as listed in Table 1 (see Supplementary Section 8).

Figure 2b shows the relationships between PHOLED LT50 and host ${R}_{{\rm{\max }}}^{-1}$ for both host families. As shown in Fig. 2b, LT50 within each host family was linearly related to ${R}_{{\rm{\max }}}^{-1}$; a smaller *R*_max_ of the host corresponded to a longer PHOLED operational lifetime. The PHOLED using DIC-TRZ, the *R*_max_ of which is smaller than that of PIC-TRZ2, showed a longer LT50 than the PHOLED using PIC-TRZ2, even though the molecular weights of the two hosts are the same. The relationships between LT50 and ${R}_{{\rm{\max }}}^{-6}$ are shown in Fig. 2c. The linear correlations between LT50 and ${R}_{\max }^{-6}$ were stronger than those between LT50 and ${R}_{{\rm{\max }}}^{-1}$ (the coefficients of determination (*r* ^2^) derived from the linear approximation method are shown in Fig. 2a and b). Figure 2d shows *r* ^2^ at each ${R}_{{\rm{\max }}}^{-x}$ as a function of a power i*n*dex *n*; *r* ^2^ was maximised at approximately *n* = 6, which corresponds to the Förster transfer model. Thus, we conclude that a requirement for the TADF host material of a stable PHOLED is a small *R*_max_.

Since the PHOLED LT50 was shown to be proportional to ${R}_{{\rm{\max }}}^{-6}$ of the host, we further examined whether LT50 is proportional to *k*_FRET_ in each host family. For this analysis, we assumed *R* to be the sum of the *R*_max_ values of the host and Ir(mppy)_3_ (see Supplementary Section 8). The estimated *R*_0_ and *k*_FRET_, summarised in Table 1, are reasonable compared to previous reports^30^. As shown in Fig. 2f and g, PHOLED LT50 was linearly related to *k*_FRET_ with relatively high *r* ^2^. In contrast, PHOLED LT50 was poorly correlated with 1*/τ*_PL_ × (*R*_0_/*R*), as shown in Fig. 2e. Such a linear relationship between lifetime and *k*_FRET_ was also observed for a different timescale (LT80; see Supplementary Section 9). Thus, the operational lifetimes of PHOLEDs using similar TADF materials as hosts were demonstrated to be proportional to *k*_FRET_. Although 27 years have passed since FRET in OLEDs became a topic of discussion^32^, and 17 years have passed since the first efficient PHOLED was reported^8^, this study represents the first observation of a strong correlation between FRET and operational lifetime in PHOLEDs. The observed *k*_FRET_-dominated lifetime is also expected to be found in other OLEDs in which FRET is possible (e.g. conventional fluorescent OLEDs and OLEDs using TADF emitters). In contrast to the *k*_FRET_-dominate lifetime, the order of estimated *k*_FRET_ is much larger than that of the RISC rate constants (*k*_RISC_) in typical TADF materials^27^. However, the observed *k*_FRET_-dominate lifetime is possible since the reported *k*_RISC_ is estimated in the TADF material itself. The *k*_RISC_ must be larger in the presence of another dopant material such as Ir(mppy)_3_ in this study^33^. Actually, the majority of the triplets in TADF-host are demonstrated to be transferred to phosphorescent dopant within 80 ns via TADF-host singlets in the PHOLED^20^. The clear relationship between *k*_FRET_ and lifetime observed in this study can be attributed to the fact that most of the excitons are transferred by FRET due to the configuration of the emitting layer, which consists of a TADF host and a small amount of dopant. This study revealed two linear relationships between lifetime and *k*_FRET_ in two host families; however, the correlation between *k*_FRET_ and the operational lifetime in the different host family remain unclear. For instance, the LT50 of the PHOLED using **2b** is almost the same as that of the PHOLED using PIC-TRZ2, whereas **2b** exhibits much larger *k*_FRET_ than PIC-TRZ2, which originates from the difference in *Φ*_PL_, spectral overlap integral and *τ*_PL_. The detailed discussion will be described elsewhere by investigating the substituent-dependent operational lifetime. At the same time, it is essential to identify donor or acceptor substituents suitable for phosphorescent hosts.

## Generality of the analysis

**Figure 3:**
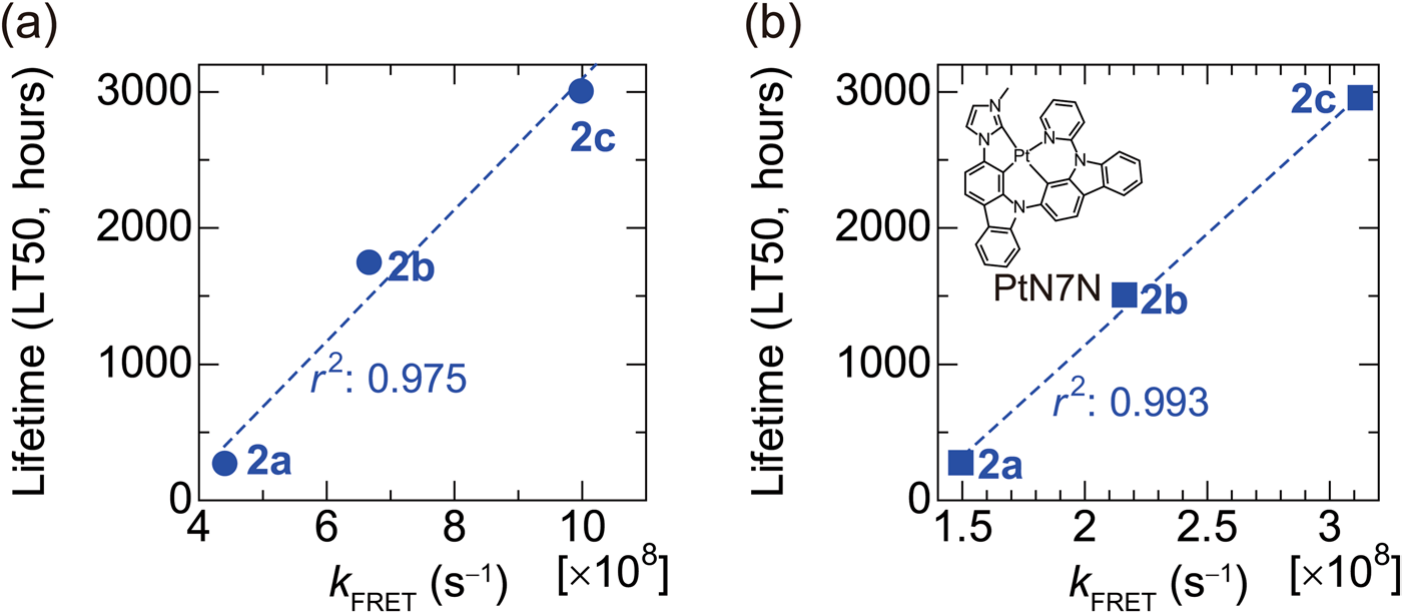
Relationship between Förster transfer rate (*k*_FRET_) and device lifetime. (**a**) *k*_FRET_ from host to Ir(mppy)_3_ versus host-dependent PHOLED lifetime with TPyQB used as the electron transporting layer. (**b**) *k*_FRET_ from host to PtN7N versus host-dependent PHOLED lifetime.

We examined the generality of the lifetime analysis by changing the electron-transporting layer (ETL) and the phosphorescent dopant (see Supplementary Section 10) and analysing again the host-dependent characteristics of PHOLEDs employing **2a**, **2b** and **2c** as hosts. Since carrier balance is thought to be one of the parameters that affects operational lifetime, we used 1,3,5-Tris(4-(pyridin-4-yl)quinolin-2-yl)benzene (TPyQB)^34^, the electron mobility of which is much higher than that of TPBi, as the ETL in the same device configuration. In addition, we used a platinum complex, PtN7N^35^, as dopant to demonstrate that the operational lifetime is dominated by FRET in PHOLEDs with non-iridium-based dopants. The chemical structure of PtN7N is illustrated in the inset of Fig. 3b. The relationships between *k*_FRET_ and the LT50s are summarised in Fig. 3. Although the driving voltage was significantly reduced by employing TPyQB as ETL (see Supplementary Section 10), the operational lifetime was nearly proportional to *k*_FRET_ (Fig. 3a). Lifetime was also proportional to *k*_FRET_ in the PHOLED using the platinum-based dopant (Fig. 3b). Thus, we can conclude that the lifetimes of PHOLEDs utilising TADF materials as hosts are dominated by FRET, independent of carrier balance and the emitter dopant.

## Conclusions

We found that the operational lifetimes of PHOLEDs are nearly proportional to the Förster resonance energy transfer rate from host to dopant. Utilising similar TADF materials as phosphorescent hosts permitted us to determine the relationship between operational lifetime and *k*_FRET_ because other than the molecular size, the photophysical properties of the hosts were similar, and almost all excitons generated by electron-hole recombination were transferred by FRET in the PHOLEDs. Our findings suggest that TADF materials with small molecular radii are suitable host materials for use in operationally stable PHOLEDs. Rapid energy transfer from the electrically excited states of host to the dopant is critical for high operational stability; at the same time, the FRET from the host singlet to the dopant, not the RISC process in the TADF host, dominates the operational stability.

One of the biggest bottlenecks to developing operationally stable PHOLEDs is eliminated in this study. The development of a novel TADF material suitable as a phosphorescent host could effectively extend the operational stability of not only blue PHOLEDs, but also PHOLEDs of other colours. In this case, an extremely small Δ*E*_ST_, which is required for efficient TADF emitters, is not necessary, and the triplet exciton of the host only have to up-convert even though Δ*E*_ST_ is relatively large. Thus, good TADF emitters and TADF materials suitable for PHOLEDs are sometimes common but basically different. The performance of PHOLEDs could be significantly improved by designing novel TADF materials suitable as phosphorescent hosts.

## Methods

### Fabrication of OLEDs

OLEDs were developed on glass substrates coated with a 100-nm-thick ITO layers. Prior to the fabrication of organic layers, the substrate was cleaned with ultrapurified water and organic solvents and treated with UV-ozone ambient. To reduce the possibility of electrical shorts within the device, Clevios HIL 1.5 was spun onto the substrate to form a 30-nm-thick layer.

The other organic layers were sequentially deposited onto the substrate without breaking the vacuum at a pressure of approximately 10^−5^ Pa. The film structures in the PHOLEDs using **2a**, **2b** and **2c** were as follows: α-NPD (20 nm)/4DBTP3Q (10 nm)/host:Ir(mppy)_3_ (25 nm)/TPBi (35 nm). After the organic layers were formed, a 0.8-nm-thick LiF layer and a 100-nm-thick Al layer were deposited as the cathodes. The devices were encapsulated using a UV-epoxy resin and a glass cover within a nitrogen atmosphere after cathode formation.

### Device characterization

The electroluminescence spectra and luminance were measured with a spectroradiometer (Minolta CS-1000). A digital source metre (Keithley 2400) and a desktop computer were used to operate the devices. We assumed that the emission from the OLED was isotropic so that the luminance was Lambertian; thus, we calculated *η*_EQE_ from the luminance, current density and EL spectra.

### Photoluminescence measurement

The 50-nm-thick organic films used for optical measurements were fabricated on clean quartz substrates by thermal evaporation. The photoluminescence (PL) spectra of the films and the transient PL characteristics were recorded using a spectrofluorometer (Horiba Jobin Yvon, FluoroMax-4). The excitation wavelength for all PL measurements was 350 or 355 nm. Sample photoluminescence quantum yield (*Φ*_PL_) was measured using a photoluminescence quantum yield measurement system (Hamamatsu Photonics, Quantaurus-QY).

## Electronic supplementary material

Supplementary_Section

## References

[1] Tang CW, VanSlyke SA (1987). Organic electroluminescent diodes. Appl. Phys. Lett..

[2] Burroughes JH (1990). Light-emitting diodes based on conjugated polymers. Nature.

[3] Forrest SR (2004). The path to ubiquitous and low-cost organic electronic appliances on plastic. Nature.

[4] Pope M, Kallmann HP, Magnante P (1963). Electroluminescence in organic crystals. J. Chem. Phys..

[5] Baldo MA (1998). Highly efficient phosphorescent emission from organic electroluminescent devices. Nature.

[6] Endo A (2009). Thermally activated delayed fluorescence from Sn^4+^–porphyrin complexes and their application to organic light emitting diodes – a novel mechanism for electroluminescence. Adv. Mater..

[7] Kondakov DY, Pawlik TD, Hatwar TK, Spindler JP (2009). Triplet annihilation exceeding spin statistical limit in highly efficient fluorescent organic light emitting diodes. J. Appl. Phys..

[8] Baldo MA, Lamansky S, Burrows PE, Thompson ME, Forrest SR (1999). Very high-efficiency green organic light-emitting devices based on electrophosphorescence. Appl. Phys. Lett..

[9] Adachi C, Baldo MA, Thompson ME, Forrest SR (2001). Nearly 100% internal phosphorescence efficiency in an organic light emitting device. J. Appl. Phys..

[10] Uoyama H, Goushi K, Shizu K, Nomura H, Adachi C (2012). Highly efficient organic light-emitting diodes from delayed fluorescence. Nature.

[11] Xiao L (2011). Recent progresses on materials for electrophosphorescent organic light-emitting devices. Adv. Mater..

[12] Zhang Q (2014). Efficient blue organic light-emitting diodes employing thermally activated delayed fluorescence. Nature Photon..

[13] Tao Y (2014). Thermally activated delayed fluorescence materials towards the breakthrough of organoelectronics. Adv. Mater..

[14] Hirata S (2014). Highly efficient blue electroluminescence based on thermally activated delayed fluorescence. Nature Mater..

[15] Tsang DP-K, Adachi C (2016). Operational stability enhancement in organic light-emitting diodes with ultrathin Liq interlayers. Sci. Rep..

[16] Zhang Y, Lee J, Forrest SR (2014). Tenfold increase in the lifetime of blue phosphorescent organic light-emitting diodes. Nature Commun..

[17] Fukagawa H (2014). Highly efficient and stable phosphorescent organic light-emitting diodes utilizing reverse intersystem crossing of the host material. Adv. Opt. Mater..

[18] Seo S (2014). Exciplex-triplet energy transfer: A new method to achieve extremely efficient organic light-emitting diode with external quantum efficiency over 30% and drive voltage below 3V. Jpn. J. Appl. Phys..

[19] Zhang D, Duan L, Zhang D, Qiu Y (2014). Towards ideal electrophosphorescent devices with low dopant concentrations: the key role of triplet up-conversion. J. Mater. Chem. C.

[20] Fukagawa H (2015). Highly efficient and stable phosphorescent organic light-emitting diodes with a greatly reduced amount of phosphorescent emitter. Sci. Rep..

[21] Zhang D (2016). Simultaneous enhancement of efficiency and stability of phosphorescent OLEDs based on efficient Förster energy transfer from interface exciplex. ACS. Appl. Mater. Interfaces.

[22] Kondakov DY, Lenhart WC, Nichols WF (2007). Operational degradation of organic light-emitting diodes: Mechanism and identification of chemical products. J. Appl. Phys..

[23] Sato K (2013). Organic luminescent molecule with energetically equivalent singlet and triplet excited states for organic light-emitting diodes. Phys. Rev. Lett..

[24] Zhang D (2014). High-efficiency fluorescent organic light-emitting devices using sensitizing hosts with a small singlet–triplet exchange energy. Adv. Mater..

[25] Fukagawa H (2016). Novel hole-transporting materials with high triplet energy for highly efficient and stable organic light-emitting diodes. J. Phys. Chem. C.

[26] Féry RCB, Vaufrey D, Doyeux H, Cinà S (2005). Physical mechanism responsible for the stretched exponential decay behavior of aging organic light-emitting diodes. Appl. Phys. Lett..

[27] Furukawa T, Nakanotani H, Inoue M, Adachi C (2015). Dual enhancement of electroluminescence efficiency and operational stability by rapid upconversion of triplet excitons in OLEDs. Sci. Rep..

[28] Dexter DL (1953). A theory of sensitized luminescence in solids. J. Chem. Phys..

[29] Förster T (1948). Zwischenmolekulare Energiewanderung und Fluoreszenz. Ann. Phys. (NY).

[30] Turro, N. J. *Modern molecular photochemistry*. 296–325 (University Science Books, Sausalito, California, 1991).

[31] Moon C-K, Kim K-H, Lee JW, Kim J-J (2015). Influence of host molecules on emitting dipole orientation of phosphorescent iridium complexes. Chem. Mater..

[32] Tang CW, VanSlyke SA, Chen CH (1989). Electroluminescence of doped organic thin films. J. Appl. Phys..

[33] Zhao Y, Zhu L, Chen J, Ma D (2012). Improving color stability of blue/orange complementary white OLEDs by using single-host double-emissive layer structure: Comprehensive experimental investigation into the device working mechanism. Org. Electro..

[34] Ahmed E, Earmme T, Jenekhe SA (2014). New solution-processable electron transport materials for highly efficient blue phosphorescent OLEDs. Adv. Funct. Mater..

[35] Fleetham, T. B. *Organic optoelectronic devices employing small molecules*. 116–122 (Arizona State University, PhD thesis, Arizona, 2014).

