# Supplementary material for: Operational lifetimes of organic light-emitting diodes dominated by Förster resonance energy transfer
Source: Sci Rep. 2017 May 11;7:1735. doi: 10.1038/s41598-017-02033-3 (PMC5431848; doi:10.1038/s41598-017-02033-3)
Supplement: Supplementary file 1 — Supplementary_Section [file 41598_2017_2033_MOESM1_ESM.pdf]

Operational lifetimes of organic light-emitting diodes dominated by Förster resonance energy transfer

Hirohiko Fukagawa*, Takahisa Shimizu, Yukiko Iwasaki, and Toshihiro Yamamoto

Japan Broadcasting Corporation (NHK), Science & Technology Research Laboratories, 1-10-11 Kinuta, Setagaya-ku, Tokyo 157-8510, Japan

*Email: fukagawa.h-fe@nhk.or.jp, TEL: +81-3-5494-3254/FAX: +81-3-5494-3297

Contents

Supplementary Section 1:

Photoluminescence measurement data of **2c-Ph** and Cz-Ph-TRZ.

Supplementary Section 2:

Thermal behaviour, energy diagram, determination of the optimal dopant concentration

Supplementary Section 3:

Comparison of device characteristics between PHOLEDs and host-only devices.

Supplementary Section 4:

Summary of PHOLED characteristics using PIC-TRZ2, DIC-TRZ and DIC-TRZ-Ph

Supplementary Section 5: The accuracy of the lifetime values (LT50s).

Supplementary Section 6: PHOLED characteristics using Cz-Ph-TRZ as host.

Supplementary Section 7: Photophysical properties.

Supplementary Section 8: Calculation of the maximum molecular radius

Supplementary Section 9: Host-dependent lifetime (LT80) versus k_{FRET}.

Supplementary Section 10:

The device characteristics of PHOLEDs using TPyQB and PtN7N.

Supplementary Section 1

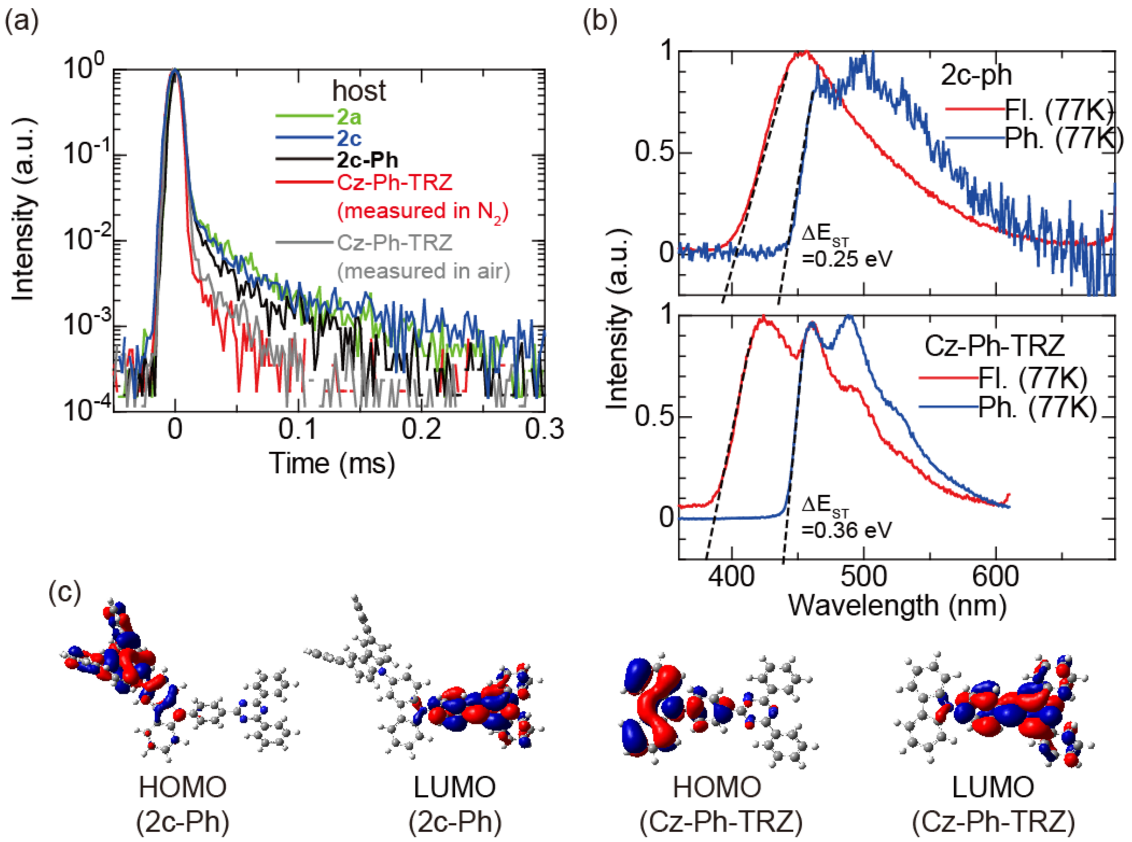

Fig. S1 (a) Transient PL curve of the 6 wt%-host-material:Ad–Cz[S1] films. (b) Fluorescence and phosphorescence spectra of 6 wt%-host-material:Ad–Cz films. Red and blue lines represent fluorescence spectra at 77 K and phosphorescence spectra at 77 K, respectively. The black dotted line represents the supporting line used to determine S_1 and T_1 energy. (c) HOMO/LUMO molecular orbital distribution obtained from the Gaussian09 program with B3LYP/6-31G(d,p) basis sets.

As can be seen in Fig. S1(a), a clear delayed fluorescence is observed from **2c-Ph**, whereas Cz-Ph-TRZ does not show a clear delayed fluorescence. That's because, the ΔE_{ST} of Cz-Ph-TRZ is relatively large as compared to other TADF materials such as **2a**, **2b**, **2c** and **2c-Ph** [see Fig. S1(b) and Table 1 in the manuscript]. The orbital overlap between HOMO and LUMO in Cz-Ph-TRZ is also relatively large as compared to other TADF materials as shown in Fig. S1(c).

Supplementary Section 2

2-1 Thermal behaviour and energy diagram

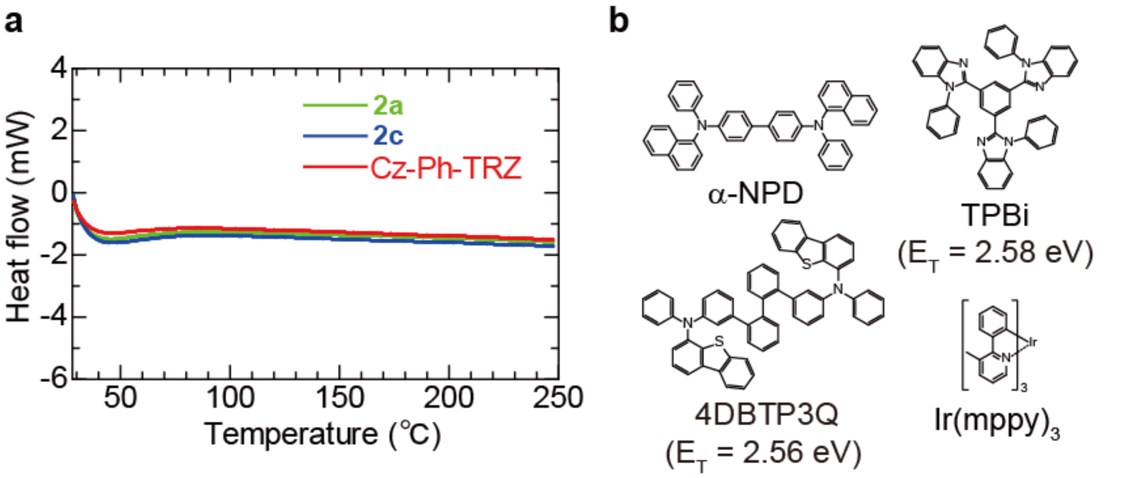

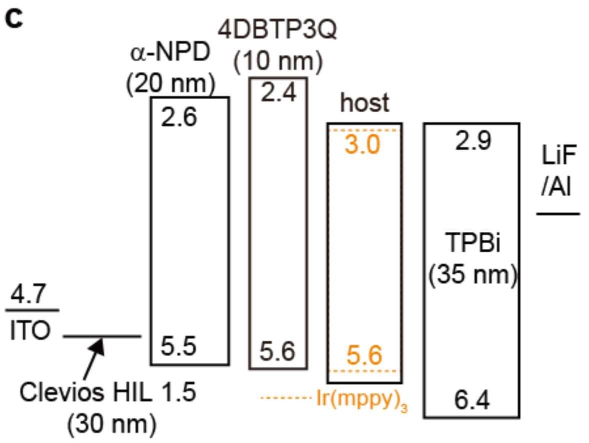

	HOMO/LUMO/Eg* (eV)	HOMO/LUMO** (eV)
**2a**	5.61/2.79/2.82	5.08/2.15
**2b**	5.66/2.78/2.88	5.16/2.11
**2c**	5.77/2.85/2.92	5.13/2.03
Cz-Ph-TRZ	5.87/2.93/2.94	5.38/1.94

*HOMO: measured by AC-3, LUMO: estimated by optical band gap (Eg)
** Calculated by Gaussian 09

Fig. S2 (a) Differential scanning calorimetry of **2a**, **2c** and Cz-Ph-TRZ. (b) Chemical structures of the materials used in this study. (c) Energy level diagram of phosphorescent organic light-emitting diodes (PHOLEDs). The HOMO level was estimated from spectroscopic measurements of photoemission in air (AC-3, Rikenkeiki). The LUMO level was estimated by subtracting the optical band gap (Eg) from the HOMO level. The optical band gap was estimated using the cut-off wavelength of the absorption peak. The HOMO/LUMO values of hosts estimated by using Gaussian 09 program are also shown for reference.

2-2 Determination of the optimal dopant concentration

Table S2: Summary of PHOLED characteristics with different dopant concentrations.

Host	Dopant concentration (wt%)	Dopant concentration (mol%)	EQE (%) [@J = 1]	Estimated LT50 (h)
**2a**	1	1.58	18.9	1,200
**2a**	3	4.69	19.5	1,260
**2a**	6	9.23	17	1,260
**2b**	1	1.15	20.4	8,600
**2b**	3	3.44	20	11,000
**2b**	6	6.87	18.7	9,500

EQE: external quantum efficiency
LT50: the time for the luminance to decay to 50% of the initial luminance of 1,000 cd m^{-2}

The optimal dopant concentration was carefully determined by fabricating PHOLEDs with different dopant concentrations as shown in Table S2. The table shows that the optimal dopant concentration in the PHOLEDs in the s-Czs host family was 3 wt%. Therefore, in these experiments, we fabricated PHOLEDs with a dopant concentration of 3 wt%.

As the molecular weight strongly depends on the host material, the mol% of the dopant in the emitting layer also depends on the host material. This causes the observed difference in the average distance of the guest [S2]. However, Table S2 shows that differences in mol% have little influence on the operational stability.

Supplementary Section 3

Comparison of device characteristics between PHOLEDs and host-only devices.

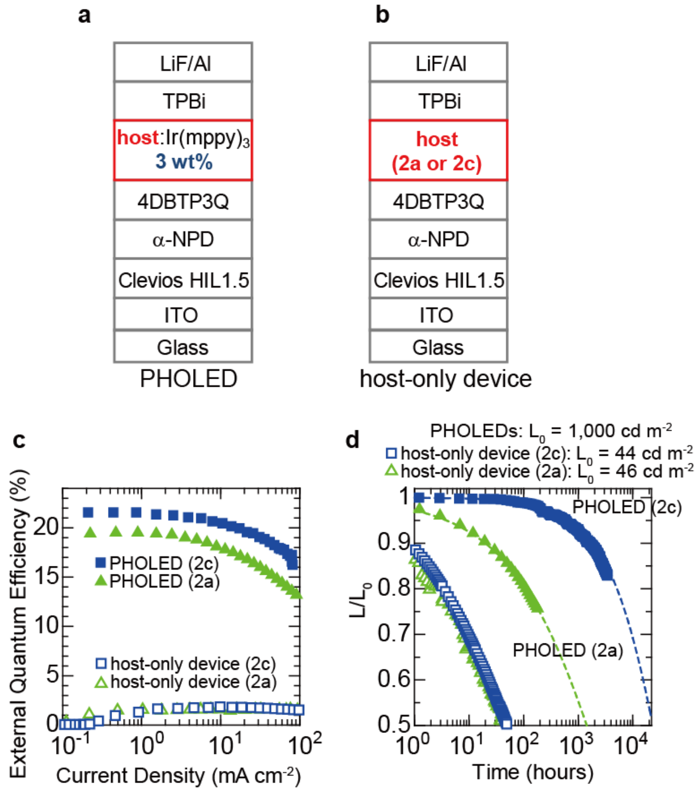

Fig. S3 (a) (b) Schematic of the device configuration. (c) External quantum efficiency–current density curves of OLEDs. (d) Luminance–time characteristics of an optimized device under a constant dc current density of approximately 1 mA cm^{-2}.

To evaluate the stability of TADF host on its own, we measured the stability of the host-only device as shown in Fig. S3d. The configurations of host-only devices are almost the same as those of PHOLEDs, except for the existence of the dopant.

As the efficiency of host-only devices is much lower than that of PHOLEDs, the stability was compared by setting the current density to approximately 1 mA cm^{-2}, which corresponds to the current density in a PHOLED at approximately L_0 = 1,000 cd m^{-2}.

Supplementary Section 4

Summary of PHOLED characteristics using PIC-TRZ2, DIC-TRZ and DIC-TRZ-Ph as the hosts.

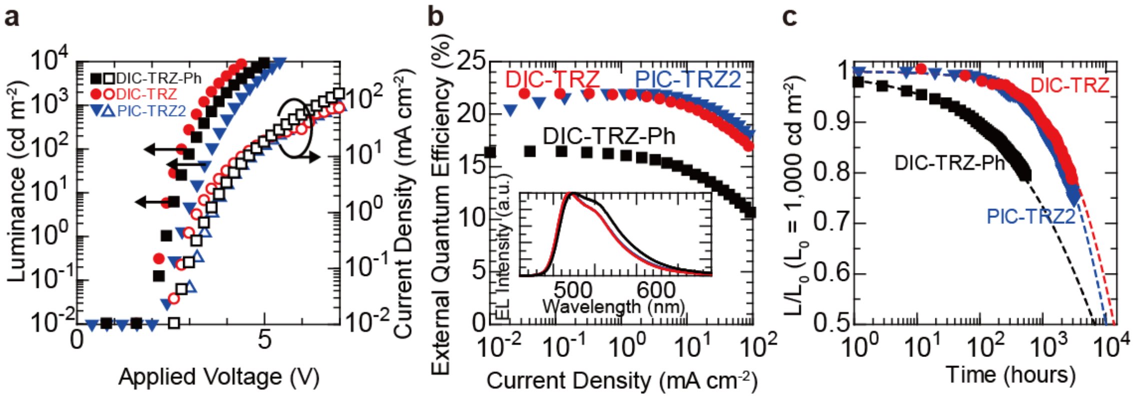

Figure S4-1 | (a) Luminance (left, filled symbols) and current density (right, open symbols)–voltage characteristics of PHOLEDs. (b) EQE–current density curves of PHOLEDs. Inset: EL spectrum of PHOLEDs. (c) Luminance–time characteristics for devices under a constant dc current with an initial luminance of 1,000 cd m^{-2}.

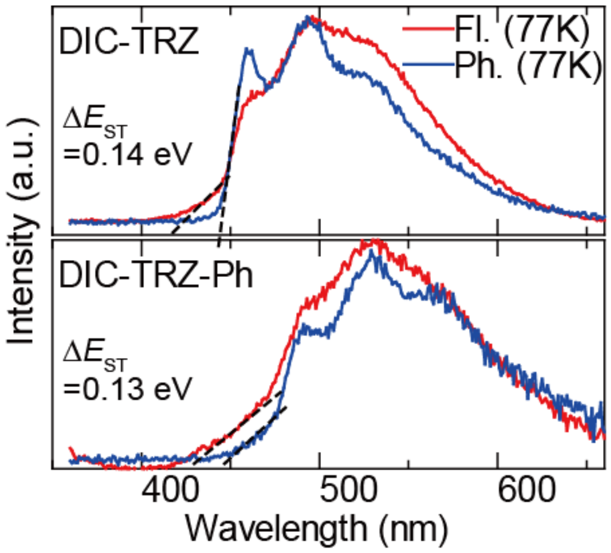

Fig. S4-2: Fluorescence and phosphorescence spectra of 6 wt%-TADF-material:Ad–Cz films. Red and blue lines represent fluorescence spectra at 77 K and phosphorescence spectra at 77 K, respectively. The black dotted line represents the supporting line used to determine S_1 and T_1 energy.

Table S4: Summary of the characteristics of PHOLEDs with different film thicknesses.

Host	Thickness of EML (nm)	Thickness of TPBi (nm)	Dopant concentration (wt%)	EQE (%) [J = 1]	Estimated LT50 (h)
DIC-TRZ	25	35	1	21.9	11,000
DIC-TRZ	35	25	1	21.8	14,000
PIC-TRZ2	25	35	1	22.1	8,200
PIC-TRZ2	35	25	1	22	10,500

The device configuration was ITO/Clevios HIL 1.5 (30 nm)/α-NPD (20 nm)/4DBTP3Q (10 nm)/host:Ir(mppy)$_3$ (25 or 35 nm)/TPBi (25 or 35 nm)/LiF/Al. As the PHOLED with a thicker EML (35 nm) exhibited a longer LT50, we set the thickness of the EML to be 35 nm in the PHOLEDs using the host family comprising ICz. The dopant concentration was set to be 1 wt% in accordance with a previous report. [Ref. 20 in the manuscript]

The *J*–*V* characteristics of PHOLEDs using the host family comprising ICz, in which the thickness of the EML is 35 nm and the thickness of the TPBi is 25 nm, are more dependent on the host than those of PHOLEDs using the host family comprising s-Czs (see Fig. S4-1a and Fig. 1c). This difference was caused by differences in the carrier transportability of the hosts. When we compare the device characteristics of these PHOLEDs, the operational lifetime of these PHOLEDs can be affected by differences in the carrier balance compared with the PHOLEDs using the host family comprising s-Czs. However, we used all the data for analysis as we needed as much data as possible to systematically understand the characteristics of host-dependent PHOLEDs.

The fluorescence and phosphorescence spectrum of DIC-TRZ and DIC-TRZ-Ph are also shown in Fig. S4-2.

Supplementary Section 5

The accuracy of the lifetime values (LT50s).

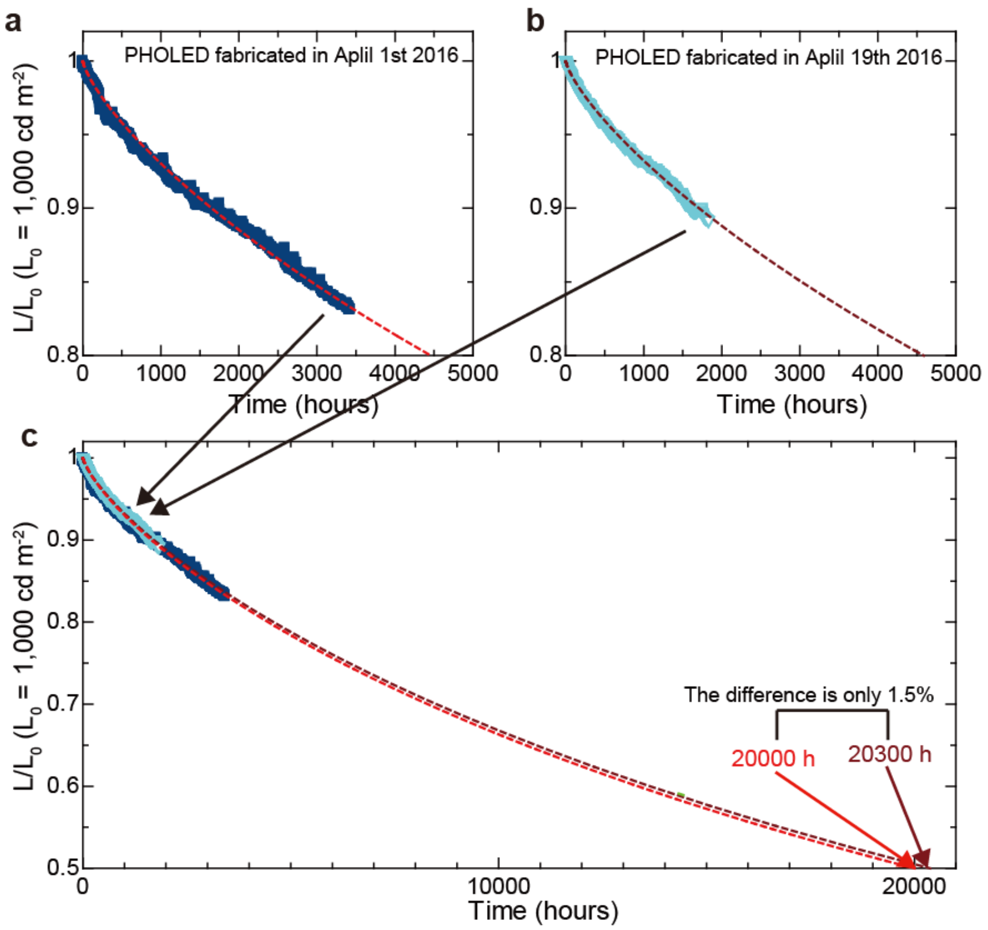

Fig. S5: Luminance–time characteristics for PHOLEDs using 2c as a host under a constant dc current with an initial luminance of 1,000 cd m^{-2}. (a) PHOLED fabricated on April 01, 2016. (b) PHOLED fabricated on April 19, 2016. (c) Comparison of the two PHOLEDs.

Almost all PHOLEDs are fabricated at least twice on different days, and the accuracy of the LT50 is checked. As shown in Fig. S5, the variation in estimated LT50 is quite small even in the most long-lived PHOLED using **2c** as a host. Thus, the accuracy of the estimated LT50 is extremely high.

Supplementary Section 6

Summary of PHOLED characteristics using Cz-Ph-TRZ, CBP as the hosts.

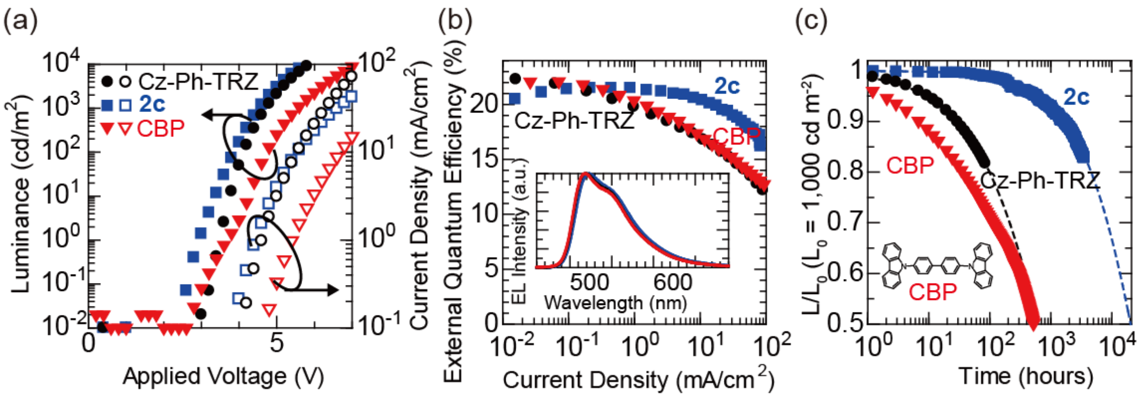

Figure S6-1 | (a) Luminance (left, filled symbols) and current density (right, open symbols)–voltage characteristics of PHOLEDs. (b) EQE–current density curves of PHOLEDs. Inset: EL spectrum of PHOLEDs. (c) Luminance–time characteristics for devices under a constant dc current with an initial luminance of 1,000 cd m^{-2}.

The operational lifetimes of the PHOLEDs using Cz-Ph-TRZ and **2c** are compared with the operational lifetime of the PHOLED using CBP, which is conventional host material consists of carbazoles. We see from Fig. S6-1(c) that the operational stabilities of PHOLEDs using CBP and Cz-Ph-TRZ are almost the same, though Cz-Ph-TRZ consists of a carbazole and a triazine. The LT50 of the PHOLED, in which the triplet up-conversion in host is impossible, are about 500 hours in this device configuration, where the dopant concentration is low. On the other hand, the LT50 of the PHOLED using **2c** is about 20,000 hours. Similar results have been reported by several groups (see Ref. 17, 19, 20 in the manuscript).

Supplementary Section 7

Photophysical properties.

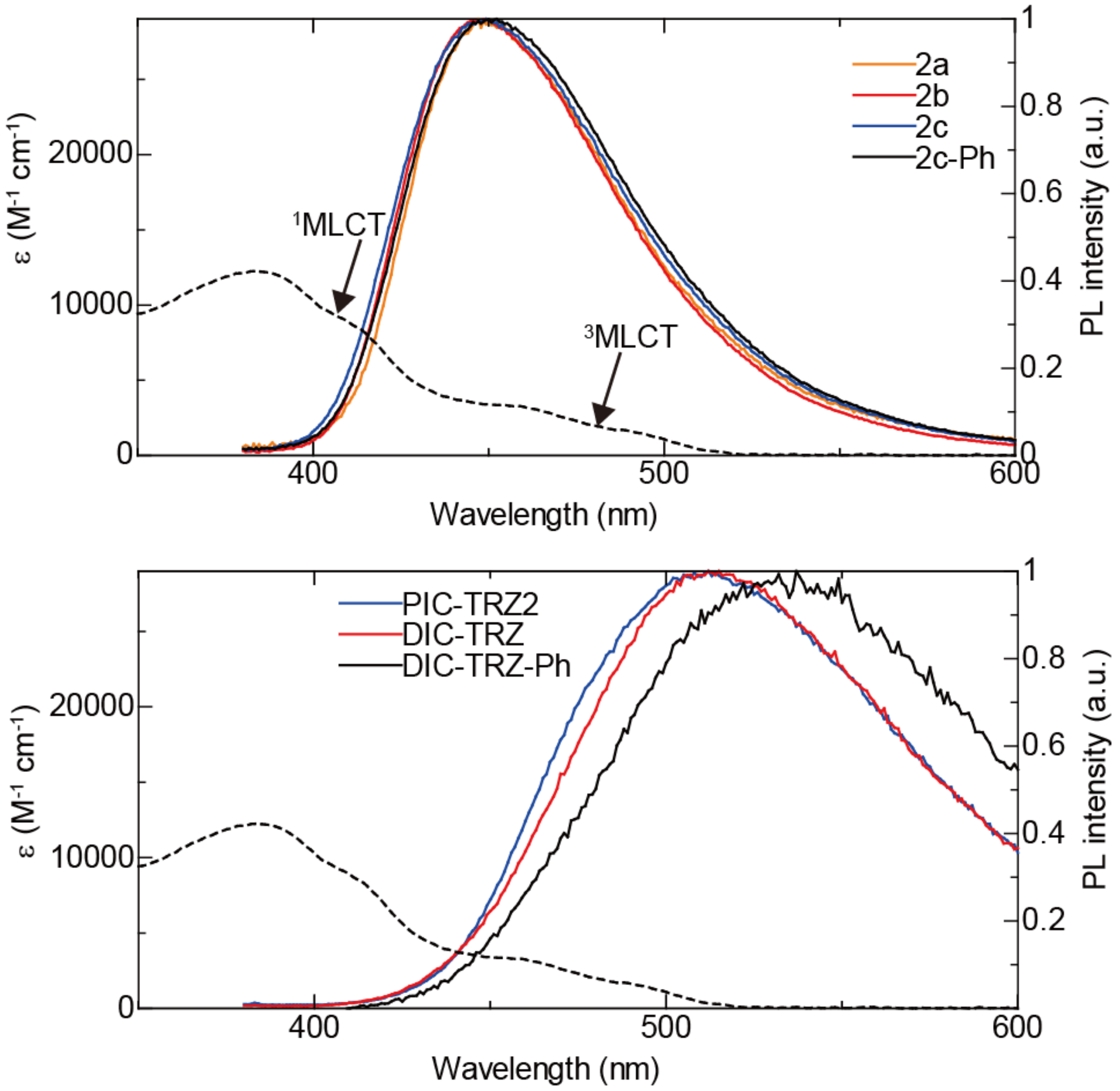

Figure S7: Absorption spectrum of $Ir(mppy)_3$ in tetrahydrofuran solution (2×10^{-5} M) (broken line) and PL spectra of 6 wt%-TADF-host:Ad–Cz film. The spectral overlap integral was calculated from this Figure.

Photophysical properties of the host that are related to k_{FRET} such as Φ_{PL} and τ_{PL} were measured in a 6-wt%-TADF-host:Ad–Cz film. Although both prompt and delayed

components were observed, we used the values related to the prompt components in the analysis. This is because the fluorescence process from S_1 to S_0 in a TADF host determines k_{FRET}.

Since the following two energy transfer processes are FRET, the Förster radius (R_0) in Table 1 was obtained from Φ_{PL} and the spectral overlap shown in Fig. S6.

S_1 (host) -> ^{1}MLCT (S_1, dopant)

S_1 (host) -> ^{3}MLCT (T_1, dopant)

.

Supplementary Section 8

Calculation of the maximum molecular radius (R_{max}) of each molecule.

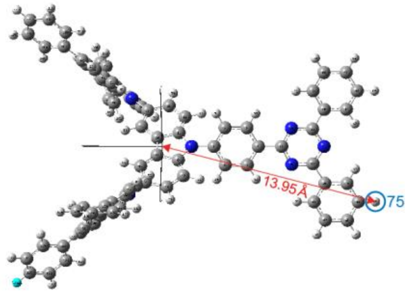

number	atomic number	x	y	z	$\sqrt{x^2+y^2+z^2}$						
1	6	−3.28787	−0.01371	−0.00438	3.287902509	71	1	−8.50331	−1.70861	3.218346	9.251128948
2	6	−3.9892	−0.60919	1.052661	4.170479338	72	1	−8.51983	1.645073	−3.22023	9.255466855
3	6	−5.37936	−0.61783	1.047549	5.515127478	73	1	−9.76324	2.643481	−5.12836	11.34058132
4	6	−6.09499	−0.02363	−0.00279	6.095036451	74	1	−12.2483	2.650222	−5.11863	13.53676771
5	6	−5.38478	0.575658	−1.0539	5.517059079	75	1	−13.4807	1.658992	−3.2018	13.95466486
6	6	−3.99456	0.576855	−1.06058	4.173018661	76	1	−12.2224	0.6637	−1.30122	12.30940858
7	7	−1.86792	−0.00876	−0.00509	1.867951439	77	6	2.200575	−5.0743	−0.49089	5.552659638
8	6	−1.05234	1.120797	−0.13186	1.54304674	78	6	2.999717	−4.07012	1.376195	5.240046129
9	6	0.306439	0.718251	−0.08422	0.78541838	79	6	1.491615	−5.41442	−1.64526	5.852154149
10	6	0.311441	−0.72023	0.07828	0.788572955	80	6	3.316873	−5.83651	−0.06372	6.713456391
11	6	−1.04451	−1.13241	0.123402	1.545508631	81	6	3.209763	−3.24511	2.483507	5.196260622
12	6	−1.40771	2.467812	−0.24427	2.851560326	82	6	3.831685	−5.19073	1.127361	6.549533508
13	6	−0.38561	3.405986	−0.33922	3.444489406	83	6	1.905097	−6.536	−2.35585	7.204071747
14	6	0.97007	3.021353	−0.29594	3.187034988	84	1	0.64092	−4.82932	−1.9772	5.257603567
15	6	1.32009	1.677849	−0.15911	2.140824438	85	6	3.710814	−6.95796	−0.79738	7.925857986
16	6	1.331759	−1.67254	0.155907	2.143657085	86	6	4.271543	−3.54865	3.328581	6.474446856
17	6	0.990975	−3.01839	0.292909	3.190380772	87	1	2.572343	−2.38947	2.677677	4.41548283
18	6	−0.36203	−3.41267	0.333299	3.447963271	88	6	4.89132	−5.47223	1.992997	7.605416524
19	6	−1.39057	−2.48187	0.235545	2.854620321	89	6	3.007656	−7.32501	−1.95187	8.155462792
20	7	1.984593	4.015256	−0.38817	4.495727708	90	1	1.346869	−6.83007	−3.23916	7.678282605
21	6	2.973749	4.089164	−1.37307	5.239251957	91	1	4.581282	−7.52964	−0.48974	8.827425302
22	6	3.795384	5.216953	−1.12205	6.548323202	92	6	5.127382	−4.65246	3.103802	7.587424318
23	6	3.276132	5.854203	0.071722	6.708940132	93	1	4.465624	−2.90032	4.177274	6.767813169
24	6	2.162076	5.085407	0.492818	5.547865046	94	1	5.516714	−6.34347	1.822657	8.602081774
25	6	3.65507	6.981922	0.803869	7.921677656	95	7	2.01206	−4.00516	0.38913	4.499013474
26	6	2.940484	7.34711	1.951815	8.150831448	96	6	3.411977	−8.51662	−2.74121	9.575420361
27	6	1.841414	6.550607	2.350769	7.199123035	97	6	3.864832	−9.68561	−2.10549	10.63865866
28	6	1.442004	5.423572	1.640878	5.846964117	98	6	3.356241	−8.5097	−4.14571	10.04321787
29	6	3.193154	3.266527	−2.48032	5.197922047	99	6	4.250943	−10.8024	−2.84481	11.95216787
30	6	4.252903	3.580631	−3.32404	6.477446331	100	1	3.88952	−9.72527	−1.02051	10.52381543
31	6	5.098026	4.692323	−3.09762	7.589666164	101	6	3.737185	−9.62792	−4.88554	11.42505563
32	6	4.853431	5.508815	−1.98624	7.60578573	102	1	3.035526	−7.60934	−4.66135	9.425739175
33	6	−7.5787	−0.02914	−0.00196	7.57875927	103	6	4.187888	−10.7799	−4.23898	12.31723185
34	7	−8.20012	−0.59066	1.044824	8.287491831	104	1	4.591293	−11.6961	−2.32946	12.77913292
35	6	−9.54264	−0.57058	1.004338	9.6122998	105	1	3.691791	−9.59498	−5.97051	11.88864549
36	7	−10.2558	−0.03977	−0.00065	10.25588013	106	1	4.486285	−11.6507	−4.81526	13.38106942
37	6	−9.54785	0.496816	−1.00624	9.613576495	107	6	6.252575	−4.93475	4.031675	8.927532428
38	7	−8.20556	0.527609	−1.04804	8.289029432	108	6	7.491711	−5.38907	3.548215	9.887245183
39	6	−10.2928	1.092206	−2.14104	10.56965891	109	6	6.110914	−4.75776	5.418875	9.452180613
40	6	−10.2816	−1.17178	2.13992	10.56714224	110	6	8.546995	−5.65946	4.417749	11.1623089
41	6	−11.6853	−1.19103	2.142174	11.93959385	111	1	7.634343	−5.50578	2.477979	9.733300125
42	6	−12.378	−1.75519	3.209915	12.90734376	112	6	7.167351	−5.02318	6.288576	10.77726663
43	6	−11.6797	−2.30573	4.287151	12.65350357	113	1	5.155028	−4.43364	5.819559	8.949788199
44	6	−10.283	−2.28991	4.291841	11.37553709	114	6	8.390759	−5.4766	5.792695	11.57381721
45	6	−9.58657	−1.7272	3.225731	10.26113357	115	1	9.497264	−6.00292	4.018619	11.93240792
46	6	−9.60321	1.654261	−3.22696	10.26506454	116	1	7.030704	−4.88504	7.357484	11.28835489
47	6	−10.3051	2.211481	−4.29232	11.38027507	117	1	9.213606	−5.68453	6.470278	12.61225074
48	6	−11.702	2.215051	−4.2868	12.65775713	118	6	6.221059	4.985833	−4.02462	8.930715986
49	6	−12.3948	1.657807	−3.20947	12.91045564	119	6	7.455798	5.450909	−3.54021	9.891126538
50	6	−11.6965	1.099162	−2.14244	11.94182125	120	6	6.081708	4.808783	−5.41205	9.455254079
51	1	−3.44008	−1.04706	1.879394	4.057412782	121	6	8.50908	5.731487	−4.40893	11.16660302
52	1	−5.92888	−1.07232	1.863069	6.306546037	122	1	7.596733	5.56809	−2.46979	9.737239915
53	1	−5.93841	1.026225	−1.86882	6.309542514	123	6	7.136162	5.084409	−6.28097	10.78084504
54	1	−3.44945	1.018505	−1.88795	4.062071426	124	1	5.128942	4.476511	−5.81348	8.952190324
55	1	−2.44596	2.778499	−0.26564	3.71124164	125	6	8.35521	5.548336	−5.78409	11.57796557
56	1	−0.62649	4.457478	−0.45307	4.524032367	126	1	9.456012	6.083224	−4.00907	11.93710331
57	1	2.366305	1.396031	−0.10013	2.749241227	127	1	7.001282	4.946032	−7.35007	11.29179971
58	1	2.376083	−1.38327	0.099491	2.751202617	128	1	9.176486	5.764249	−6.46108	12.6166558
59	1	−0.59566	−4.46578	0.447373	4.52748739	129	6	3.328758	8.544375	2.740558	9.570665187
60	1	−2.42662	−2.79993	0.25481	3.713895984	130	6	2.356041	9.38451	3.30941	10.2260526
61	1	4.490389	7.590421	0.470388	8.831724022	131	6	4.680887	8.872613	2.939758	10.45352293
62	1	1.308471	6.812059	3.259621	7.66429209	132	6	2.720629	10.50834	4.049064	11.58542248
63	1	0.6061	4.821183	1.979513	5.246869014	133	1	1.304466	9.166851	3.147455	9.7795328
64	1	2.563868	2.405181	−2.67574	4.417906061	134	6	5.046198	9.998891	3.675098	11.78763254
65	1	4.453915	2.934765	−4.173	6.772303279	135	1	5.450983	8.22283	2.534738	10.1859239
66	1	5.470293	6.385935	−1.81497	8.602232382	136	6	4.067881	10.82227	4.234811	12.31270833
67	1	−12.2155	−0.76069	1.301017	12.3081276	137	1	1.94958	11.1459	4.472532	12.16698275
68	1	−13.4639	−1.76593	3.202879	13.95179172	138	1	6.098339	10.2273	3.820446	12.50532569
69	1	−12.2217	−2.74521	5.119547	13.53200783	139	1	4.352347	11.69851	4.809872	13.376584
70	1	−9.73679	−2.71668	5.127786	11.33488528						

Fig. S8: Optimized molecular structure of **2a** and the coordinates of each atom. The distance between each atom and the original point was calculated using the Pythagorean theorem.

Table S8: Summary of the estimated maximum molecular radius (R_{max}) of each molecule.

Molecule	R_{max} (nm)
**2a**	1.51
**2b**	1.31
**2c**	1.22
**2c-Ph**	1.42
PIC-TRZ2	1.02
DIC-TRZ	0.93
DIC-TRZ-Ph	1.29
Ir(mppy)$_3$	0.77
PtN7N	0.93

The distance between the centre of mass and the farthest hydrogen atom was calculated in the optimized structure, and the van der Waals' radius of the hydrogen atom was added to it. The coordinates of the optimized structure were obtained from the Gaussian09 program with B3LYP/6-31G(d,p) basis sets. One example of the results we calculated is shown in Fig. S8; the distance between the centre of mass and the farthest hydrogen was calculated in **2a**. As the original point of the coordinates corresponds to the centre of mass in this calculation, the distance between the centre of mass and the farthest hydrogen atom can be calculated as shown in Fig. S8.

Supplementary Section 9

Host-dependent lifetime (LT80) versus k_{FRET}.

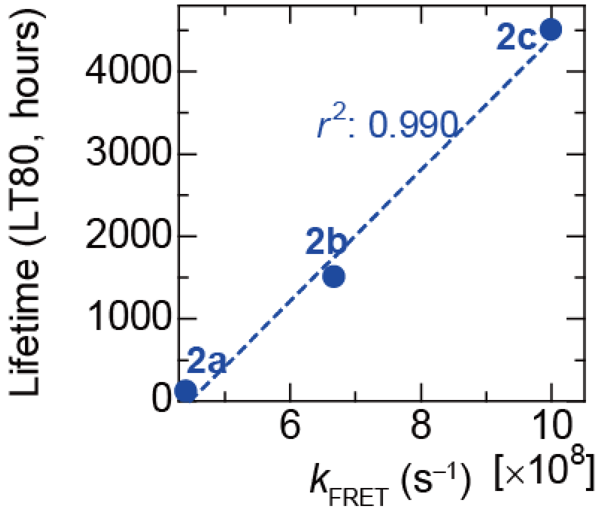

Fig. S9. Host-dependent lifetime (LT80) versus k_{FRET}.

Supplementary Section 10

The device characteristics of PHOLEDs using TPyQB as the electron transporting layer (ETL) (Fig. S10-1 **a**, **b** and **c**) and PtN7N as the emitter dopant (Fig. S10-1 **d**, **e** and **f**).

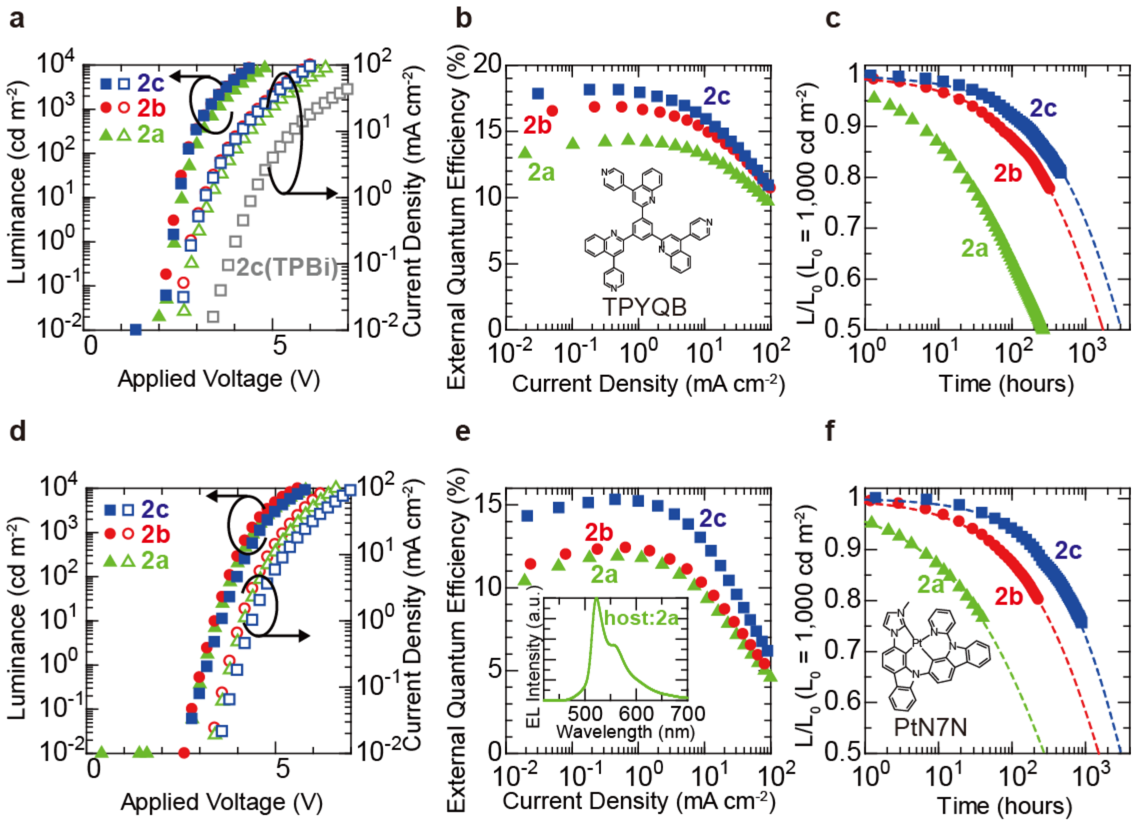

Fig. S10-1. The device characteristics of PHOLEDs using TPyQB as the ETL (Fig. S10-1 **a**, **b** and **c**) and PtN7N as the emitter dopant (Fig. S10-1 **d**, **e** and **f**). The device configuration was ITO/Clevios HIL 1.5 (30 nm)/α-NPD (20 nm)/4DBTP3Q (10 nm)/host:dopant (3 wt%, 25 nm)/ETL (35 nm)/LiF (1 nm)/Al (100 nm) (see also Table S10).

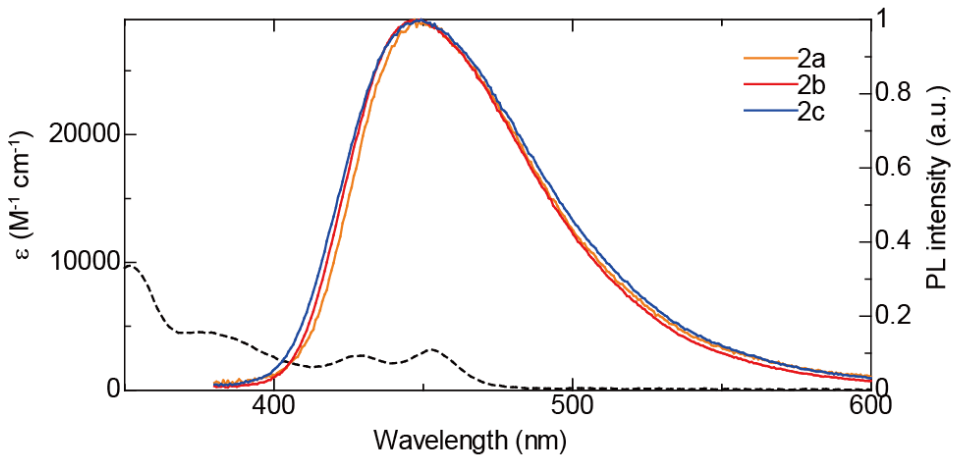

Fig. S10-2. Absorption spectrum of PtN7N in tetrahydrofuran solution (2 × 10^{-5} M) (broken line) and PL spectra of 6 wt%-TADF-host:Ad–Cz film.

Table S10: Summary of the configurations of PHOLEDs, PHOLED performances, parameters related to k_{FRET} and the calculated k_{FRET}. The spectral overlap is shown in Fig. S10-2.

Host	Dopant	ETL	PHOLED performances		R_0 (nm)	k_{FRET} (10^8 s^{-1})
			EQE* (%)	LT50 (hours)		
**2a**	$Ir(mppy)_3$	TPyQB	14.3	265	-	4.42
**2b**	$Ir(mppy)_3$	TPyQB	16.7	1,700†	-	6.68
**2c**	$Ir(mppy)_3$	TPyQB	17.9	3,000†	-	10.0
**2a**	PtN7N	TPBi	11.8	270	2.43	1.5
**2b**	PtN7N	TPBi	12.3	1,500†	2.41	2.2
**2c**	PtN7N	TPBi	15.2	3,000†	2.43	3.1

*Measured at 1 mA cm^{-2} (corresponds to luminance of approximately 1,000 cd m^{-2}).
†Estimated lifetime. The accuracy of the value is relatively high (see Supplementary Section 5)
R_0: Estimated critical distance for the concentration quenching (Förster radius).
k_{FRET}: Estimated Förster transfer rate from host to $Ir(mppy)_3$.

The data used for analysing the lifetime with respect to k_{FRET} is also shown (Table S10). The shorter lifetime of TPyQB-based PHOLEDs compared to TPBi-based PHOLEDs may be because of the poor stability of TPyQB [Ref. 25 in the manuscript].

Reference

S1. Fukagawa, H., Watanabe, K., Tsuzuki, T., Tokito, S. Highly efficient, deep-blue phosphorescent organic light emitting diodes with a double-emitting layer structure. *Appl. Phys. Lett.* **93**, 133312 (2008).

S2. Kawamura, Y., Brooks, J., Brown, J. J., Sasabe, H., Adachi, C. Intermolecular Interaction and a Concentration-Quenching Mechanism of Phosphorescent Ir(III) Complexes in a Solid Film. *Phys. Rev. Lett.* **96**, 017404 (2006).
